# Cerebrospinal fluid (CSF) shunting and ventriculocisternostomy (ETV) in 400 pediatric patients. Shifts in understanding, diagnostics, case-mix, and surgical management during half a century

**DOI:** 10.1007/s00381-016-3281-1

**Published:** 2016-10-29

**Authors:** A. Henriette Paulsen, Bernt J. Due-Tønnessen, Tryggve Lundar, Karl-Fredrik Lindegaard

**Affiliations:** Department of Neurosurgery, Oslo University Hospital, Oslo, Norway

**Keywords:** Pediatric CSF diversion, Shifts in case-mix and management

## Abstract

**Objective:**

To characterize shifts from the 1960s to the first decade in the 21st century as to diagnostics, case-mix, and surgical management of pediatric patients undergoing permanent CSF diversion procedures.

**Methods:**

One hundred and thirty-four patients below 15 years of age were the first time treated with CSF shunt or ETV for hydrocephalus or idiopathic intracranial hypertension (IIH) in 2009–2013. This represents our current practice. Our previously reported cohorts of shunted children 1967–1970 (*n* = 128) and 1985–1988 (*n* = 138) served as backgrounds for comparison.

**Results:**

In the 1960s, ventriculography and head circumference measurements were the main diagnostic tools; ventriculoatrial shunt was the preferred procedure (94 %), neural tube defect (NTD) was the leading etiology (33 %), and overall 2-year survival rate was 76 % (non-tumor survival 84 %). In the 1980s, computerized tomography (CT) was the preferred diagnostic imaging tool; ventriculoperitoneal shunt (VPS) had become standard (91 %), the proportion of *NTD* children declined to 17 %, and the 2-year survival rate was 91 % (non-tumor survival 95 %). Hydrocephalus caused by *intracranial hemorrhage* had, on the other hand, increased from 7 to 19 %. In the years 2009–2013, when MRI and endoscopic third ventriculocisternostomy (ETV) were matured technologies, 73 % underwent VPS, and 23 % ETV as their initial surgical procedure. The most prevalent etiology was *CNS tumor* (31 %). The proportion of *NTD* patients was yet again halved to 8 %, while *intracranial hemorrhage* was also reduced to 12 %. In this last period, six children were treated with VPS for *Idiopathic Intracranial Hypertension* (IIH) due to unsatisfactory response to medical treatment. They all had headache, papilledema, and visual disturbances and responded favorably to treatment. The 2 years of survival was 92 % (non-tumor survival 99 %). In contrast to the previous periods, there was no early shunt related mortality (2 years). *Aqueductal stenosis* was a small but distinctive group in all cohorts with 5, 6 and 3 % respectively.

**Conclusions:**

The case-mix in pediatric patients treated with permanent CSF diversion has changed over the last half-century. With the higher proportion of children with CNS tumor patients and inclusion of the IIH children, the median age at initial surgery has shifted substantially from 3.2 to 14 months. Between the 1960s and the current cohort, 2 years of all-cause mortality fell from 24 to 8 %. Prolonged asymptomatic periods, extending 15 years, were relatively common. Nevertheless, 18 patients experienced shunt failure more than 15 years after last revision, and first-time shunt failure has been observed 29 years after initial treatment. This underscores the importance of life-long follow-up.

## Introduction

Despite significant advances that have transpired in the treatment of hydrocephalus during the last six decades, most neurosurgeons still regard the management of pediatric hydrocephalus as a challenge. Albeit hydrocephalus has been clinically recognized since antiquity; the current understanding of its pathophysiology is mainly a product of the 19th and 20th centuries [1]. In a classical study, largely valid even today, Key and Retzius in 1875 suggested that the cerebrospinal fluid (CSF) was secreted by the choroid plexus, flowed out of the ventricular system, and was reabsorbed through the subarachnoid villi and pacchionian granulations [1]. Once hydrocephalus was recognized as a mechanical hydraulic disorder, it was realized that theoretically treatment might be through any of the following three means: reducing CSF production by deactivating the choroid plexus, reopening intracerebral blocked fluid pathways with a bypass or a surgical removal of the causative lesion, or shunting CSF into body cavities of normally low pressure using a valved tubing system.

Today’s treatment has resulted from discoveries within numerous fields underlining an inextricable mutual bond between basic science and clinical therapy.

Before CSF shunting became common, i.e., in the 1940–1950s, the mortality rate was about 50 % (about one in two children died), and many of the survivors has debilitating deficits [2]. Technological breakthroughs made silicon shunts possible, providing biocompatibilities [1, 3]. At the same time, improved valve technology emerged which explains why between 1956 and 1961 valved shunts had become standard of care [1].

It soon transpired, however, that in most cases shunt surgery could relieve the acute life-threatening disorder while converting it to a chronic condition. Nowadays, mortality is mostly due to the underlying cause of hydrocephalus.

For a period, ventriculoatrial shunts (VAS) were standard, superseded by ventriculoperitoneal shunts (VPS) from about 1980.

In our department, Arne Torkildsen introduced ventriculocisternostomy in 1937 using rubber catheters. These proved to be effective in selected cases [4]. Following the advent of reliable valved shunts, ventriculocisternostomy was performed only occasionally, but since the 1990s advanced neuroendoscopic techniques have resulted in a renaissance of ventriculostomy.

We have chosen a procedure-centered focus rather than concentrating on a more or less well-defined disease entity. The aim of the present work is thus to portray some long lines from the evolving practice of CSF diversion procedures. To this end, we present representative snapshots from three different epochs spanning one-half century of pediatric CSF diversion surgery from the 1960s until today.

## Material and methods

All patients under 15 years of age who underwent initial treatment for hydrocephalus and IIH with either a prosthetic shunt system or endoscopic third ventriculocisternostomy (ETV), in the Neurosurgical Department, Oslo University Hospital, Oslo, Norway, between 01 January 2009 and 31 December 2013, were retrospectively analyzed. The patients were selected from surgical protocols according to the NOMESCO (Nordic Medico-Statistical Committee) classification of surgical procedures (NCSP) [5]. The codes selected were the following: ventriculostomy (AAF00), ventriculoperitoneal shunt (AAF05), lumboperitoneal shunt (AAF10), ventriculoatrial shunt (AAF15), shunt of intracranial cysts to peritoneum (AAF40), and other shunt operation (AAF99) (Subduroperitoneal shunt), i.e., all types of prosthetic shunts, and ETV procedures were included. The diagnose of hydrocephalus was based on increased head circumference, typical clinical signs of raised intracranial pressure, and enlargement of the ventricular system on neuroradiological imaging; and in ambiguous cases, intracranial pressure (ICP) recording was used. All the IIH patients had papilledema, and the indication for shunting was stationary or progressive visual field defects despite medical treatment, intractable headache, or both.

The following details were ascertained from the case records: name, age, gender, indication of initial permanent CSF diversion, later CSF diversion procedures, hardware removal with subsequent re-implantation, surgical complications (e.g., shunt infection), survival, and cause of death. CSF diversion procedures due to presumed treatment failure were performed in the presence of moderate to severe clinical symptoms of shunt dysfunction, in most cases, supported by radiological findings. In some patients with vague clinical symptoms and/or non-conclusive radiological findings, ICP monitoring was used to verify the presence of drainage failure, (over- or under drainage) or not, prior to revision.

Two previous cohorts of children, both including children younger than 15 years of age, initially shunted for hydrocephalus in the same institution during the years between 1967–1970 (*n* = 128) and 1985–1988 (*n* = 138) and formed the basis for our comparison [6, 7].

The results from these three time epochs were used to describe our experience as well as shifts in diagnostic work-up, case-mix, and surgical management during half a century.

## Statistics

These cohorts include children considered to be in need for CSF diversion referred to our department in the actual time periods. There are obvious differences as to case-mix and surgical approach. Besides, we assume that there are unknown confounders. Statistical probability analysis may therefore seem inappropriate and has therefore not been performed.

## Results

### Recent cohort

In this first section, we present results from children treated with CSF shunt or ETV in the calendar years 2009–2013. We identified 134 patients (81 males, 53 females) first time treated with CSF shunting or ETV in our department in 5 years, 2009 to 2013. The patients were followed between 2 and 7 years or until death - follow-up was complete in all cases. Last available follow-up was at an average of 8 years 4 months (median 6 years 10 months, range 2.5 to 20.5 years).

The cause of intracranial hypertension is summarized in Table 1. The most common underlying condition was *CNS neoplasm* (31 %). The vast majority of children in need for CSF diversion in the *Hemorrhage* (12 %) group were infants, born preterm with intraventricular or/and intracerebral hemorrhage. Neural tube defects (NTD) accounted for 8 % of the children. The group, here referred to as *Other malformation* (22 %), included a wide range of causes, see Table 1. Patients with IIH accounted for six patients (4 %). Other children without any obvious cause to their elevated ICP were denoted as *Unknown* (17 %).

**Table 1:** The underlying cause for permanent CSF diversion in 134 pediatric patients during the calendar years 2009–2013

Cause		No. of patients (%)
CNS Neoplasm		42 (31)
Intracranial neoplasms		
Supratentorial loc.	19	
Infratentorial loc.	17	
Supra- and infratentorial loc.	2	
Intraspinal neoplasms	1	
NF type 1/TS	3	
Hemorrhage		16 (12)
IVH and/or ICH	13	
SAH/other vascular malformations	2	
Acute subdural hemorrhage	1	
Neural tube defects (NTD)		11 (8)
Myelomeningocele		
Lumbosacral loc.	8	
Thoracal loc.	1	
Encephalocele	1	
Anencephaly	1	
Aqueductal stenosis		4 (3)
Other malformations		29 (22)
Syndromal associated HC	11	
Intracranial cysts		
Fossa posterior cysts	3	
Arachnoidal cysts	3	
Chiari 1	5	
Craniosynostosis, non-syndromatic	3	
Dermal sinus tract	1	
Septum pellucidum cysts	1	
Other	2	
Postinfectious		3 (2)
Unknown		23 (17)
Idiopatic intracranial hypertension (IIH)		6 (4)
Total		134 (100)

The mean age at first-time treatment (prosthetic shunt or ETV) was 3 years 11 months (median 1 year 2 months, range 1 day to 14 years 12 months). In 43 (32 %) patients, first treatment was performed during their first 6 months of life; and in 64 (48 %) patients, within the first year of life. Time of initial treatment varied considerable within the different subgroups; whereas, the mean age was 6 years 11 months (median 6 years 11 months) in the *Tumor* group and 6 months (median 10 days) in the *NTD* group. If excluding the patients with *Tumor* and *IIH*, the mean age at first surgical treatment for hydrocephalus was 2 years 1 month (median 7 months).

Within 2 years of initial surgery (prosthetic shunt or ETV), more than half of the patients (52 %) needed to undergo at least one re-do. Repeat surgery was more frequent in the *Hemorrhage* group (mean 2.4), lower in the *Tumor* group (mean 1.1), while no revisions were performed in the three patients with hydrocephalus due to *Infection*. In total, 59/134 (44 %) patients did not require further hydrocephalus-related surgery, and 51/118 (43 %) of whom were alive at follow-up.

### Patients initially treated with a prosthetic shunt

In 103 (77 %) patients, a prosthetic shunt was placed to treat the child’s intracranial hypertension (98 VPS, 2 CPS, 1 LPS, 2 S-DPS). All of whom had clinical symptoms indicating raised intracranial pressure and the vast majority presented with ventriculomegaly on neuroimaging. In some patients ICP recording was used as an adjuvant diagnostic procedure prior to permanent CSF diversion. Furthermore, continuous over-night ICP-recording was used for all the 6 IIH patients to reveal mean ICP pressure and mean wave amplitudes. The mean age at shunt insertion was 3 years 8 months (median 11 months, range 1 day to 14years 7 months). The proportion of children without any revision of initial shunt between 1 and 2 years was 53 % (52/98) and 44 % (41/94), respectively. In times of shunt failure, eight patients were treated with ETV. Two of them did not need further treatment, while six later received a prosthetic shunt. At follow-up (2–7 years), 89 out of 103 initially shunted children were still alive. Twenty-five of these (28 %) had no revision of their prosthetic shunt during the study period.

Idiopatic intracranial hypertension (IIH) was the cause of shunt placement in six patients (three boys, three girls). The mean age at shunt insertion (VPS) was 9 years (median 9 years 8 months, range 4 years to 12 years 3 months). Due to further worsening of vision despite shunt placement, orbitotomy was performed bilaterally in one male patient followed by 11 shunt revisions during follow-up. In the remaining five patients, only one revision was registered. Except from one female patient who in the following has been diagnosed with a hereditary eye condition, the remaining patients reported relief of headaches, improved vision and resolution of chocked disks.

### ETV as initial treatment

In 31 (23 %) patients, ETV was the preferred choice of initial surgery. Almost half of them (15 children) had intracranial tumor, of which ten in the posterior fossa. The patients being primarily treated with ETV tended to be slightly older than the individuals first time treated with a prosthetic shunt system with a mean age of 4 years 7 months (median 1 year 6 months). Two patients, both with malignant tumor, died within 2 years of follow-up.

The proportion of children with no re-dos (repeat ETV or conversion to prosthetic shunt) at 1 and 2 years was 67 % (20/30) and 62 % (18/29), respectively. Mean revision-free time in those 11 patients who needed further revisions was 5 months (median 2 months, range 1 day to 2 years). In one patient, a re-stoma was successfully performed, while in the remaining ten patients, VPS was placed.

### Shunt related morbidity and mortality

Two hundred and thirty-three surgical procedures (shunt revisions, ETVs, and re-ETVs), with a range of 1–14 procedures for each of the affected children, were recorded. Shunt infection did occur in 14 cases in 12 (9 %) patients, with an incidence of infection per procedure of 3.8 %.

Of the 134 patients evaluated, 16 (12 %) died during follow-up. Analyses revealed survival rates of 98.5, 95.5, and 91.8 at 0.5, 1, and 2 years, respectively (form initial treatment until death), see Table 2. In all 16 cases, the most probable cause of death was due to their underlying condition; 15 patients died due to malignant CNS tumors, and one patient shunted in palliative care died due to severe malformations (anencephaly).

**Table 2:** Survival rates in pediatric patients treated with permanent CSF diversion in three different time epochs

	No. of patients (non-tumor)	Overall survival rates (non-tumor)	
Time epoch		At 1 year	At 2 years
1967–1970	128 (103)	84 (88)	76 (84)
1985–1988	138 (115)	93 (96)	91 (95)
2009–2013	134 (92)	96 (100)	92 (99)

Excluding the tumor patients revealed, survival rate was at 100 % at 1 year and 99 % (91/92) at 2 years.

### Patients with CNS tumor

In total, 42/134 (31 %) patients had hydrocephalus associated with CNS tumor. This subgroup differs from the others due to the high mortality rate of 15/42 (36 %), and age at initial treatment. We found a predominance of boys with a male/female ratio of 24/18 = 1.33. There was an even distribution of patients with tumor located supra- and infratentorially (21/42, 50 %), and there was a predominance of high-grade WHO III/IV (24/42, 57 %) tumors. Except from eight patients, all with low-grade tumors, the remaining 34 patients had adjuvant chemo- and/or radiotherapy.

Endoscopic third ventriculocisternostomy was selected as initial intervention for the child’s hydrocephalus in 15/42 (36 %) instances. In all 15 patients, nine of whom had an infratentorial location of their tumor, intracranial hypertension was relieved *before* tumor surgery. Preoperative ETV was persistently successful in 12 patients, and three patients were in need for re-surgery due to their hydrocephalus; in one patient, a re-stoma was subsequently performed, while in the remaining two patients, a VP-shunt was inserted.

Prosthetic shunt systems (VP) were inserted in the latter 27 tumor patients, *before* (14 patients) or *after* (13 patients) tumor resection.

### Comparison of results in the three different cohorts—from the 1960s, 1980s, and the twenty-first century

**Fig. 1:**
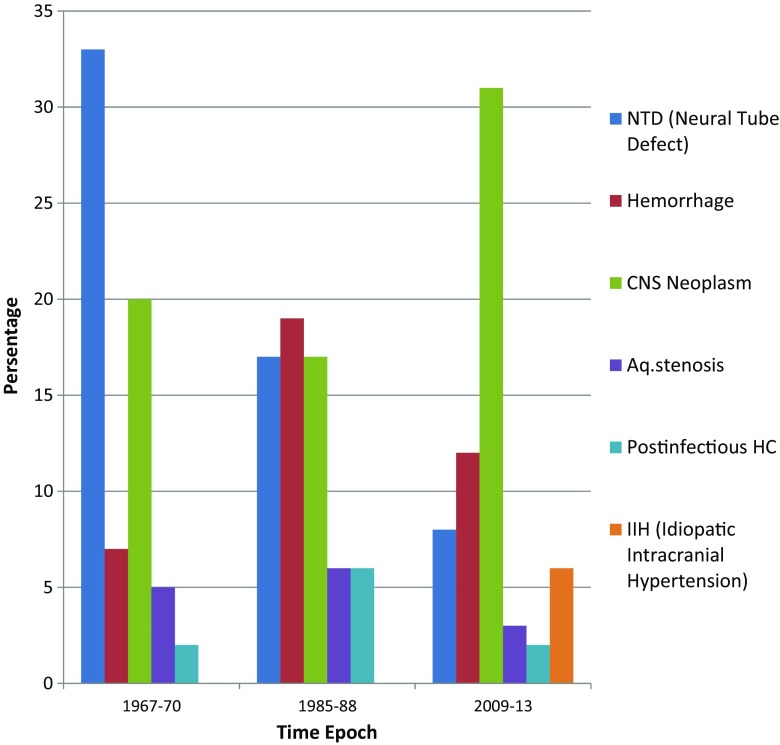
Case-mix in children less than 15 years of age are in need for permanent CSF diversion (prosthetic shunt or ETV) due to intracranial hypertension in three different time epochs

The cause of the hydrocephalic state raised intracranial pressure in pediatric patients differs greatly in the three cohorts studied. Table 3 gives a brief overview of our major findings. Males outnumbered females in all three cohorts with a male/female ratio of 1.2, 1.9, and 1.5, respectively. Figure 1 demonstrates the etiological factors for each given time epoch. In the 1960s, NTD was accounting for one third of the children requiring shunt insertion [7]; in the 1980s, the proportion was nearly halved to 17 percentages [6], and in the twenty-first century, the proportion of children was only 8 %. Hemorrhage was the cause of shunting in 7 % in the 1960s, increased to 19 % in the 1980s, and reduced to 12 % in the years 2009–2013 [6, 7]. The most common distinct etiological group in the third cohort was CNS neoplasm (31 %) which accounted for 20 and 17 % in the first and second period. Aqueductal stenosis was a small but distinctive group in all the cohorts with 5, 6, and 3 %, respectively. Consequently, there were divergent results regarding age at initial treatment in the three cohorts. The median age in the most recent cohort was 14 months, compared to 6 months in the 80s, and 3.2 months in the 60s.

**Table 3:** Comparison of three cohorts of children treated with permanent CSF diversion in different time epochs

			Time epoch		
	Variable		1967–1970	1985–1988	2009–2013
Cohort (no. patients)			128	138	134
Follow up (years)			42–45	20–24	2–7
Median age at follow up (years)			44.6	23.6	6.8
Diagnostic work-up^a^			Ventriculography	CT	CT/MR
Demography	Median age, initial treatment (months)		3.2	6	14
	sex-ratio (M/F)		1.2	1.9	1.5
Etiology (%)	Determined	Neural tube defect	33	17	8
		Hemorrhage	7	19	12
		CNS neoplasm	20	17	31
		Aqueductal stenosis	5	6	3
		Postinfectious	2	6	2
		Other malformations^b^	9	18	22
		IIH	0	0	4
		Trauma	0	3	0
	Undetermined	Unknown^b^	24	14	17
Event-free survival (%)^c^		at 1 year	64	61	56
of the shunt		at 2 years	42	40	48
Mortality (%)	Overall	at 1 year	16	7	4
		at 2 years	24	9	8
	Non-tumoral	at 1 year	12	4	–
		at 2 years	16	5	1
Mortality (no)	Shunt-related	at 2 years	4	1	–

^a^Besides head circumference measurements and clinical signs, this was the most common used neuroimaging technique at time of inclusion

^b^Patients amounting for the *Congenital communicating HC*-group in the cohort from the 1960s were included in the *Unknown*-group, while patients shunted due to posterior fossa cysts/porencephaly, multiple malformations, chromosome defect, Arnold-Chiari malformation, and CNS disease in the late 1960s were included in the *Other malformation*-group

^c^Elective shunt revisions are excluded

**Fig. 2:**
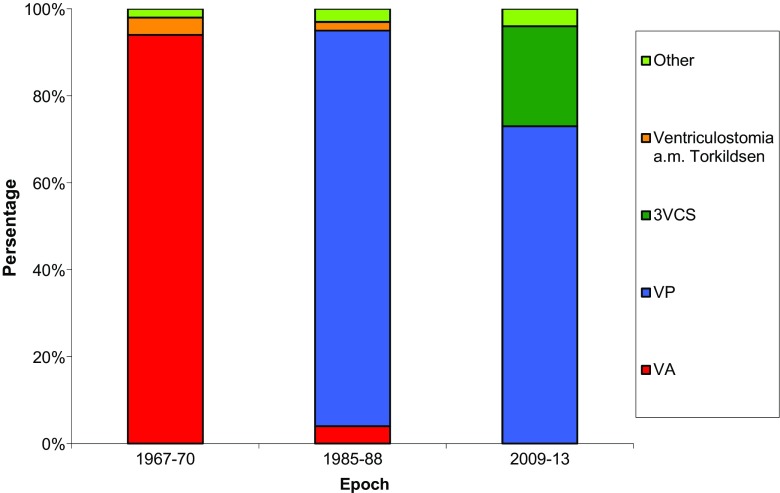
CSF-shunting in pediatric hydrocephalus patients changing practice patterns 1967–2013

Our findings concerning preferred initial surgical treatment in children with HC raised intracranial pressure are illustrated in Fig. 2. VA shunt was the initial surgical procedure in 94 % of the cases in our study conducted in the 1960s. In the 1980s, VP shunts was the most common procedure and accounted for 91 %, and the proportion treated with VA shunts had diminished to 4 %. In the third cohort from the twenty-first century, 73 % underwent VPS and 23 % ETV as the first surgical treatment due to raised intracranial pressure.

Event-free survival rate of the primary shunt at 2 years in children treated in the late 60s was 34 % (33 patients of 97 still-alive patients). Excluding elective elongations of the atrial catheters, 42 % of the patients had not experienced shunt failure. In the cohort treated in 1985–88, event-free survival rate after 2 years was 40 % (50 patients of 125 still-alive patients), and in the cohort from 2009 to 2013, event-free survival rate of initial treatment (prosthetic shunt or ETV) at 2 years was 48 %.

The overall and non-tumor survival rates (between 1 and 2 years) in the three different time epochs are presented in Table 2.

A long interval between episodes of shunt failure was seen in a number of cases. Fifteen patients in the cohort from the late 1960s and three patients in the cohort from the 1980s experienced stable shunt function for 15 years or more, where after shunt failure occurred. Additionally, one patient experienced first-time shunt failure no less than 29.2 years after initial treatment. This patient had intracranial hemorrhage following intake of amphetamine.

## Discussion

This report includes *all* pediatric patients who received permanent CSF diversion during 2009–2013. All patients presented with a variety of typical clinical features indicative of raised intracranial pressure, and the vast majority had mild to severe ventricular enlargement on neuro-imaging. The six patients diagnosed with IIH presented with small or “normal sized” ventricles according to their age. The inclusion of IIH patients may be found controversial by some colleagues claiming that these patients differ from the traditional concept of hydrocephalus. Still, the need for CSF diversion was evident in both entities; and since, there was no children with IIH (Pseudotumor cerebri or benign intracranial hypertension) in our previous two cohorts in the years 1967–1970 and 1985–1988, we found them most proper to include in this historical review, not least, as a supplement for describing differences in case-mix throughout time.

In Norway, as in Sweden and other western countries, the incidence of NTD has decreased over the past decades. Nikkilä et al. did perform a population-based study in Sweden covering the years from 1973 to 2003 showing that the rate of spina bifida in newborns diminished gradually from 0.55 to 0.29 per 1000 [8]. In theUSA, between 1970 and 1989, the neural tube defect rate declined from 1.3 to 0.6 per 1000 births [9]. The decline is probably, to a great extent, a consequence of prenatal ultrasound screening and selective abortion of affected fetus. Better nutritional intake, usage of folic acid supplement before and during early pregnancy, amniocentesis, and screening for alpha-fetoprotein may also have been contributing factors [9, 10]. The gradual decreasing number of shunted individuals with NTD in our three cohorts from the 1960s, the 1980s and further, or until today corroborate with their findings [6, 7].

The advances in neonatal intensive care seen during the 1970s and 1980s reduced perinatal mortality, and more very preterm infants with IVH survived and developed hydrocephalus [11]. Consequently, we encountered more preterm newborns with IVH in need for CSF diversion in the 1985–1988 compared to in the 1967–1970. Despite a continued reduction in perinatal mortality throughout the 1990s, the prevalence of hydrocephalus in infants born very preterm decreased. This was most likely due to improved peri and neonatal care, described by Fernell et al. [12]. The reduced number of newborns in the most recent cohort in need for CSF diversion due to IVH corroborates with those findings.

Summarizing the proportion of patients with *NTD*s and *Hemorrhage* in need for permanent CSF diversion in 1967–1970 and 2009–2013, we find that this proportion has been halved during a time span of 50 years, from 40 to 20 %. At the same time, in the cohort from the twenty-first century, we found a higher proportion of hydrocephalus associated with CNS tumor compared with our previous series [6, 7]. Some studies have found a gradual increase in the incidence of pediatric brain tumors during the last decades [13]. In the USA, a similar increase in childhood brain tumors was reported occurring in the mid-1980s and was explained by an increase, primarily in the detection and reporting of low-grade astrocytomas secondary to improved diagnosis and registration [14]. Likewise, an increase in pediatric brain tumors has also been found in Norway in recent years [15].

During the past five decades, there has been a national increase in the number of hospitals with a neurosurgical care unit, and changes has been made regarding the national geographical distribution of pediatric patients with brain tumor. Therefore, the higher proportion of CNS tumor in this cohort does not necessarily indicate a higher annual incidence of pediatric brain tumors. Yet, our studies only concern *hydrocephalic* CNS tumor patients. Due-Tønnessen et al. performed a study on 100 consecutive children surgically treated for low-grade cerebellar astrocytomas where 15 % experienced a persistent hydrocephalic problem after resection of the tumor [16].

Comparing the three cohorts did reveal a step-wise increase in age at initial treatment. The changing case-mix represented by fewer patients, with NTD and preterm neonates with IVH in need of CSF diversion, explain some of this difference. Further, patients with CNS neoplasm and IIH contribute to increasing the average age. Since the decision for CSF diversion is made by the surgeon on an individual basis, a change in policy over time cannot be fully excluded.

### Surgical management

Since the late 1950s, hydrocephalus treatment became more standardized, and shunts into the venous system (usually into the right atrium) were the preferred procedure. Not surprisingly, 94 % of the shunts in 1967–1970 were VAS. Nevertheless, the unavoidable problem of children with longitudinal growth caused gradual retraction of the atrial catheter and serious side effects such as thromboembolic events, pulmonal hypertension, shunt nephritis and sepsis, which led to a negative trend [17–19]. In the 1970s, the use of VP shunts steadily gained ground, and it became the most commonly used variety of shunt. Figure 2 clearly illustrates this shifting practice, and in 1985–1988, we found that 91 % was VPS. Progress in neuroendoscopic technique has been one important contributor to the fact that ETV has gained widespread acceptance as an effective way to manage hydrocephalus in selected pediatric patients. Kulkarni et al. [20] did show that age, etiology, and the presence of previous CSF shunt were each important and independent factors in predicting success of ETV in children and thereby have proposed an ETV success score calculated on these three strata. However, Buxton et al. [21] reported a 23 % success rate in a group of children (with communicating and non-communicating HC) less than 1 year of age (mean age 3.7 months) and suggested that, despite the fact that the majority fail, ETV should be considered as the first-line treatment for hydrocephalus in this age group to spare some individuals the added morbidity of having a shunt (avoid future shunt-related morbidity). Drake et al. conducted a multicenter study in Canada including 368 pediatric HC patients (34 % brain tumors) and found a 1-year ETV success rate of 65 %, when successful outcome hinged on the absence of further CSF diversion procedures [22].

In our most recent cohort, 38 % of the ETV patients had undergone subsequent surgical procedure for CSF diversion within 2 years. This does not imply a 2-year success rate of 62 %, since 15 out of 31 were tumor patients who underwent initial ETV before tumor removal. Previous reports have demonstrated that the cure rate of hydrocephalus in children with tumor in the posterior fossa was 59 % after tumor resection alone [23]. In our series, two of these patients died within the 2-year period, while some of the other 13 most probably were cured for their hydrocephalic problem after tumor removal. On the other hand it seems clear that the ETV eliminated the acute hydrocephalic problem before tumor surgery.

In some cases of secondary hydrocephalus, improvement may appear after treatment of primary cause due to the reestablishment of CSF pathways. This may cause overestimation of ETV success rate and should therefore be taken into account when comparing ETV outcome.

Nevertheless, treatment with valved shunts has remained the most common procedure in recent years. Our 2009–2013 cohort reveals that VP shunts are used in almost three fourths of the children.

### Re-do surgeries

In the most recent cohort, the tumor patients had a lower revisions rate compared to the other etiological subgroups. This could be due to the average shorter observational time caused by deaths shortly after initial treatment, and the fact that some patients, especially those with infratentorial tumors, were cured for their hydrocephalic condition after resection of its origin (tumor).

Pediatric shunt failure rates may be one of several parameters used to evaluate the treatment standard. When VAS was the treatment of choice, elective revisions for lengthening of the atrial catheter were needed in times of growth of the child, whereas the necessity of elective elongations became rarer/non-existing after the introduction of VPS. Moreover, there are essential differences in terms of shunt hardware and the availability of diagnostic tools in our three series which make the comparison challenging. Consequently, comparing revision rate and event-free shunt survival to investigate whether any progress has been made in preventing shunt failure over the past decades are contaminated by differences in practice. Besides, the high early mortality rate in our study on children shunted in the late 1960s, and the high proportion of successfully procedures with CSF diversion among the tumor patients prior to resection of tumor in the posterior fossa in the most recent cohort may both influence our results. Thus, considering our results regarding shunt survival rate at 2 years of 42, 40, and 48 %, in the first, second, and third period, respectively, the results has not clearly improved.

Similar findings were presented by Stein et al. [24] who performed a structured search in the literature to determine whether failure rates of hydrocephalus shunts had fallen in the period from the late 60s and up until the twentieth century, excluding elective lengthening of VAS, where they concluded that no convincing evidence in a reduction of pediatric shunt failure rates were found.

In our two previous series (1967–1970 and 1985–1988), both with more than 20 years of observation, more than one out of ten patients being shunt dependent at follow-up experienced shunt failure after 15 years or more. This substantiates the need for lifelong follow-up.

### Early mortality

Due to limited observational time in our latest study, this makes us unable to calculate mortality rates extending 2 years of duration. Since our results from the late 1960s did reveal rather discouraging findings concerning overall short-term survival with only 76 % of the patients surviving at 2 years, our two more recent cohorts from the 1980s to the twenty-first century did present with a higher survival rate of 91 and 92 %, respectively.

The shunt-related mortality rate of 3 % at 2 years in patients treated with VAS in 1967–1970, combined with the fact that there were a high proportion of children dying of unknown cause, made us to propose that treatment with shunt in the 1960s was associated with a higher risk of mortality due to treatment compared with more recent years. No early shunt-related mortality was registered in the most recent cohort.

### Follow-up

CSF diversion for hydrocephalus matured in the late 1950s and became established during the 1960s. Although shunt failure was a recognized problem even in the early phase, the most common practice was to wait until blockade of the shunt forced intervention [25]. The awareness upon preventive strategies to avoid complications to shunt treatment and improve outcome in shunted individuals became clearer with time. Becker et al. introduced a program for elective revisions to maintain shunt function in 1960, i.e., elongation of the atrial catheter in growth periods of the child [25]. Besides, Foltz emphasized the importance by periodic and thorough evaluation of the functioning status of the shunt (irrespective of symptomatology) by measurement of ventricular pressure, comparison of ventricular size, psychometric tests, and physical status of the flushing device [26].

Although the majority of the patients in the cohort from the 1960s were routinely followed, there were some exceptions. The higher early mortality rate in the 1967–1970 cohort could reflect that at the time, management and follow-up had yet to reach maturity.

Still, even today, more than 50 years later, there is no consensus regarding the optimal mode of controlling these patients in regard to frequencies of visits and imaging.

The complexity and rarity of the condition make the follow-up a specialist’s role. The role of brain imaging is much debated. It is important to obtain a baseline cerebral imaging to provide adequate comparison when suspicion of shunt malfunction arises. Some authors advise a clinical visit in older children being asymptomatic of shunt failure every second year [27]. In our institution, newly shunted infants have scheduled control at 1 month, and then at 2 to 6 months. Thereafter, in the absence of symptoms of possible shunt malfunction, we currently perform controls at 1 to 2 years, with the frequency decreasing as the child grows.

### Limitations of the study

The retrospective nature of this study poses several methodological limitations. First, patient selection was determined by the treating surgeon. Varying individual thresholds for determining both the need for operative intervention for hydrocephalus, decision upon prosthetic shunt or ETV, and the need for repeat intervention could have an impact on patient selection, choice of treatment, and the observed failure rates. Second, comparing results of CSF diversion in pediatric patients in three different time epochs will inevitably also reflect the inherent differences in practice patterns and thereby influence the results. Due to the complexity of confounding factors, we have refrained from using multivariable statistical analyses in this series. Third, because the most recent series were performed relatively recently, the length of follow-up is rather short, thereby limiting our ability to make comparisons extending 2 years of duration. Taken together, these limitations force us to be cautious in the interpretation of our results and emphasize the empirical nature of our study.

Evolvement is continuous, and understanding current practices in order, hopefully, to foster future improvement calls for knowing the long lines leading from the practice of the past to that of the present.

## Conclusions

The case-mix in pediatric patients in need for permanent CSF diversion has evolved during half a century. There has been a marked decline in the proportion of infants with neural tube defects and IVH in need for treatment due to intracranial hypertension. Almost one third of the children in need for CSF diversion in 2009–2013 were diagnosed with CNS tumor. Patients tend to be older at initial treatment in the twenty-first century compared to 50 years ago. Although the need for re-do surgery does not differ notably in the three time epochs, early-mortality (at 1 and 2 years) seems to have been lowered. Severe shunt failure may take place at any time, even after prolonged periods of stable shunt functioning, indicating the need of life-long follow-up.

## Abbreviations

ETV: Endoscopic third ventriculocisternostomy
IIH: Idiopatic intracranial hypertension
VPS: Ventriculo peritoneal shunt
VAS: Ventriculo atrial shunt
CSF: Cerebrospinal fluid
CPS: Cysto peritoneal shunt
LPS: Lumbo peritoneal shunt
S-DPS: Sub-dural peritoneal shunt
NTD: Neural tube defect
ICP: Intracranial pressure
